# The electronic media and the study profile of the surgical resident

**DOI:** 10.1590/0100-6991e-20212941

**Published:** 2021-07-10

**Authors:** JULIA REIMBERG, LUIZ ROBERTO LOPES, SILVIA MARIA RICETO RONCHIM PASSERI, FÁBIO HUSEMANN MENEZES

**Affiliations:** 1 - Faculdade de Ciências Médicas da Universidade Estadual de Campinas (UNICAMP), Departamento de Cirurgia - Campinas - SP - Brasil

**Keywords:** Education, Medical. Teaching. Internship and Residency. Education, Distance, Educação Médica, Ensino, Internato e Residência, Educação à Distância

## Abstract

**Objective:**

this study analyzed the surgical resident’s study profile by assessing the use of electronic media. 44.76% of the physicians agreed on participating.

**Methods:**

observational, cross-sectional non-controlled study. Statistical analysis was performed using Pearson’s correlation coefficient and the significance level for the statistical tests was p <0.001.

**Results:**

87.2% of the residents believed that it is interesting to use e-learning together with the classical theoretic classes. 45% of the interviewed claimed to spend more than 3 hours on the Internet daily.

**Conclusion:**

residents recognize the importance of technology for education, but not as a way of replacing the traditional teaching methods.

## RESUMO

**Objetivo:**

este trabalho analisou o perfil de estudo dos residentes em Cirurgia, caracterizando o uso das mídias eletrônicas, por meio de questionário padronizado, com índice de resposta de 44,76%.

**Métodos:**

estudo descritivo, observacional transversal, sem grupo controle. O coeficiente de correlação de Pearson foi usado para a análise estatística e o nível de significância adotado foi de p <0,001.

**Resultados:**

observou-se que 87,2% dos residentes julgaram interessante associar o e-learning às aulas teóricas. Sobre o tempo despendido no uso de tecnologia, 45% referiram usar internet mais de 3 horas por dia.

**Conclusão:**

a partir dessa pesquisa, pode-se observar que os residentes atribuem importância à tecnologia em sua formação, todavia sem substituir o ensino tradicional.

## INTRODUCTION

Medical Residency combines hands-on patient care and teaching-learning activities balanced, in order to provide solid knowledge and better clinical and practical skills during specialization^1^ ^-^ ^2^.

According to Botti and Rego^3^, the teaching-learning process must rely on metacognition, among other components, which is one of the bases of constructivism. This metacognition is defined as the individual ability to become a responsible and active seeker of knowledge, based on daily clinical practice provided by the residency program. The learner should reflect on these opportunities and look for consolidating information to achieve a better professional performance^3^.

It is observed that the classical theoretic teaching is left to a second level among the daily activities. Emphasis is directed to patient care and research^4^. The Brazilian National Residency Committee published the 02/2006 resolution, on May 17th, 2006, which establishes a limit of 10-20% of the total residency time for activities directed to theory^5^.

Throughout the residents’ specialization, the large volume of information that must be passed on the students during such a limited time period poses a challeng for medical institutions. Thus, new forms of knowledge transmission must be considered^6^ ^-^ ^7^.

E-learning was first used for medical training in 1961, and since then, it has advanced as an important tool in Medical Education^8^ ^-^ ^9^. E-learning allows for a wider availability of contents for the students anywhere, at any time, especially with the introduction of notebooks, tablets, and smartphones^10^.

E-learning is defined, in a broader way, as a learning tool based on the internet^11^. In Medicine, it encompasses three different systems - online lectures, virtual patients and discussion forums^12^. With e-learning, it is possible to create interactive material, simulations, and access to a larger range of knowledge, providing active and independent learning^13^.

Internet based learning also allows for instant access to information regarding clinical decision making, and a more concise presentation of the content^14^. Another advantage is to allow study flexibility regarding frequency, duration, and content, that may be directed according to the need^8^.

Despite all these benefits, there is a constant conflict between the traditional teaching methods and e-learning, regarding when and how to use them, and as an alternative on how to think Medical Education^11^. Nowadays, e-learning is well accepted as a supportive technology and not as a substitute of traditional teaching^14^.

Taking these e-learning aspects into consideration, the purpose of this paper was to assess how our surgical residents used electronic media and their practices regarding the study of theory.

## METHODS

The data were collected in the year 2019, and this project was undertaken as the scientific initiation of one of the authors (JR). 

This is an observational, transverse study which included interviews with the regularly attending residents of the General and Specialty Surgery Programs of the Hospital of Clinics of the University of Campinas. A standardized questionnaire was used for this purpose (Attachment 1). 

After approval from the local Ethics Committee (CAE: 92664218.0.0000.5404), all residents from the General and various Specialty (Head and Neck, Digestive, Trauma, Pediatric, Plastic, Thoracic, Vascular, Coloproctology, Intensive Care and Urology) Programs were invited to participate. The participants’ answers of the standardized questionnaire were acquired during an interview with one of the authors (JR). All participants signed the informed consent form. The study intended to interview all 105 surgical residents of the Department of Surgery, but It was only possible to interview 47 (44.76%) residents. The reason why some residents decided not to reply to the invitation was not recorded and could become the reason for a future investigation. 

Data were registered in a spreadsheet (Microsoft Excel) and statistical analyses were performed with SPSS - Statistical Package for the Social Sciences, and GraphPad Prism software. Descriptive statistics are presented in the tables. For correlations, the Pearson test was applied. It was adopted the level of significance of p<0.001.

## RESULTS

Data were separated in four categories: 1) use of digital resources, 2) management of time, 3) study strategies for future exams, and 4) interest in having available digital content.

1) Use of digital resources:

36 (76.6%) of the residents indicated that it is preferable to use notebooks and desktop computers, and 11 (23.4%) preferred the use of tablets. 41 (87.2%) of the residents believed it would be more interesting to have activities as e-learning as well as to have alternating on site lectures. Only 2 (4.2%) said that they would prefer to have only e-learning. Only one (2.1%) would prefer on site lectures alone, and 3 (6.5%) did not answer this topic. Thirty seven (78.7%) reinforced that on-site classes were important and 9 (19.1%) classified the on-site classes as indifferent, irrelevant or extremely irrelevant.

When asked about the need of exams to promote learning, 25 (53.2%) reported them as necessary to support study theory, 21 (44.7%) reported they studied regardless of tests, and 1 (2.1%) did not answer.

Regarding the use of printed material (books) over digital, 29 (61.7%) reported a preferential use of the former. The same residents reported a preference for handbooks specifically written with the purpose of teaching residents over classical textbooks.

Forty-one (87.2%) residents reported good access to scientific journals, six (12.8%) had no access. Also, forty-one (87.2%) residents do not regularly go to the Institution Library to access the journals, while six (12.8%) do so, and one of the latter reported having no access to scientific journals (Table 1).

**Table 1:** Surgical residents’profiles .

Preferred study method	Study support	Study strategies for future residency exams
76.6% computers	61.7% preferably read printed textbooks	87.2% have attended a preparatory course for the current residency program exams
87.2% e-learning activities alternated with on-site classes	61,7% preferably read handbooks written for residents	66% intend to attend a preparatory course for the next step (specialty entrance exams)
53.2% believe that formative tests at regular intervals help the study organization	87.2% have access to scientific journals	
78.7% reported the importance of on-site classes	87.2% do not frequently go to the Institution’s library	

2) Management of time:

 Twenty-four (51%) residents indicated to read less than 3 hours a week regarding the study of surgical theory. Thirty-one (66%) spent less than 3 hours a week studying in Medical books, and 37 (79%) reported to dedicate less than 3 hours a week to read scientific papers. Twenty-one (45%) residents spent more than 3 hours a day to navigate on the internet, read e-mails, etc.

There is a positive correlation between the time spent reading surgical topics and the reading of Medical textbooks (Table 2).

**Table 2:** Average time spent in the study of Surgery, textbook reading, scientific papers reading and use of internet.

	> 5 h	4-5 h	3-4 h	1-3 h	< 1 h
Weekly hours spent in the study of surgical theory*	9 (19%)	4 (9%)	10 (21%)	21 (45%)	3 (6%)
Weekly hours spent reading medical textbooks	7 (15%)	1 (2%)	8 (17%)	16 (34%)	15 (32%)
Weekly hours spent reading scientific papers*	1 (2%)	2 (4%)	7 (15%)	22 (47%)	15 (32%)
Daily hours spent to navigate on the internet (all purposes)	0	13 (28%)	13 (28%)	17 (36%)	4 (9%)

Positive correlation R=0.8074 , p<0.001, Pearson test.

3) Study strategies for future exams:

It was observed that 87.2% of the participants attended some sort of preparatory course to take the exams for the current residency program, and 66% intend to attend a preparatory course to take the exams for a specialty residency program.

4) Interest in having available digital content:

The University uses the Moodle Platform to support the management of the digital content, with 57.4% of the participants stating they had already used it during their undergraduate or the current residency programs, and 53.1% of them defining it as an interesting platform (Table 3).

**Table 3:** E-learning thoughts among surgical residents.

E-learning thoughts
68% believe that a synchronous video classes are not more interesting than an on-site class
74.4% regularly look for basic clinical discussion forums and videos to help understand a theoretical concept
91.5% believe that e-learning is an efficient learning tool
57.4% have used the Moodle platform during their undergraduate or residency programs
53.1% believe that the Moodle platform is interesting

Thirty-two (68%) of the residents considered the traditional on-site classes more important than the online activities (Table 3). Finally, 25 (53.1%) of the residents frequently use a mobile phone to check on instructions in order to prescribe medications, while 19 (40.4%) use it to review disease clinical presentations and differential diagnosis.

## DISCUSSION

We were able to show, in the current study, residents (87.2% of the interviewed subjects) are inclined to support the use of e-learning alongside with on-site classes. Contradictorily, despite a big interest in the use of digital technology, 61.7% of the residents declared a preference for a paper-based study. Yamsom et al.^15^, in 2018, demonstrated that 57% of the students also preferred the use of printed material over digital. Leven et al.^16^, in the year 2000, stated that 90% of the medical students preferred printed material.

We have also been able to see that 87.2% of the participants do not regularly make use of the resources available in the Institution’s Library. Oussalah et al.^17^, in 2015, stated that only 46% of the residents accessed the traditional textbooks, because they preferred to read scientific journals. In the present research, it was found that 61% of the residents preferred the use of concise manuals over classical textbooks. 

Edson et al.^18^, in 2015, found that 77% of the participants dedicated less than seven hours a week to read medical literature. They also reported that 94% of the participants used UpToDate as their main source of knowledge. In the present paper, we observed the same, since only 15% of the residents dedicated more than five hours a week studying.

Regarding the need for motivational tests to enhance the study habits, the results were similar. It is interesting to highlight that tests were viewed as ranking instruments and a way of promoting competition in the academic environment. However, when adequately taken, they can be an effective method to promote auto-evaluation and reflection, in particular in terms of acknowledging the mistakes to enhance the teaching-learning process. Larsen et al.^19^, recommend that tests should be carried out weekly or monthly to stimulate a regular rhythm of reading, and organization.

In a residency setting, evaluation is also an important instrument to highlight topics in need of more knowledge, thus decreasing the educational gap, and as a result, protecting patients. Such assessment may be used in different ways, besides the common written tests, to better evaluate competencies^20^.

As mentioned, 87.2% of the residents valued the use of e-learning reinforced by on-site classes. Silva et al.^21^, in 2011, demonstrated that medical students who underwent additional e-learning performed better after having taken exams.

This method allows the access of digital content at any time and place, with better image resolution and lower cost. It also allows for interaction among users and instant feedback^21^ as well as repetitive viewing to enhance the comprehension and acquisition of important techniques through didactic videos^22^. This is specifically important in teaching Surgery, and an effective e-learning method for residents^23^.

Digital technology contributes to fast and repetitive access to information, and although it is a welcome additional learning tool, it does not minimize the importance of the teacher in the learning environment. We believe that e-learning may improve learning goals along with other educational methodologies^24^.

There is a possibility to better explore the digital resources focused on theoretical knowledge acquisition in the surgical programs, including surgical technique video content, clinical cases discussion forums, frequent formative evaluation tests, and a methodology to help with time organization and self-evaluation of performance. This study can contribute to the implementation of new and more efficient methodologies of study, that are better suited to the current surgical residents’ study habits.

This study has limitations such as the lack of sample size calculation and the inclusion of only half of the Institution’s residents. The latter might be explained by the fear of being identified since some programs had only one resident.

## CONCLUSION

This group of surgical residents frequently consumes digital content from the internet, and spends significant part of their time navigating its content, either for entertainment or study purposes. The residents study less than seven hours a week, and although they are acquainted with new technologies, they prefer printed study materials. They believe that e-learning resources are complementary and do not replace traditional on-site classes.

## ATTACHMENT 1

Questionnaire:

1) Use of digital resources

1. How many hours a day do you spend with your mobile phone to surf on the internet, read e-mails, etc.?

( ) < 1h ( ) 1-3 hs ( ) 3- 4 hs ( ) > 4 hs

2. Would you rather study on:

( ) notebook or desktop computer ( ) tablet ( ) mobile phone

3. I usually search on forums and online videos to better understand a concept that I am not sure of it.

( ) yes ( ) no

4. I frequently use mobile phone apps to expeditiously clear doubts:

( ) Clinical presentation and differential diagnosis ( ) bullarium and instructions for use ( ) risk stratification tables ( ) I don’t use my apps for this purpose

2) Management of study time

5. How many hours a week (mean) do you use to study theoretical surgical concepts?

( ) < 1h ( ) 1-3 hs ( ) 3- 4 hs ( ) 4-5 hs ( ) > 5 hs

6. What is the percentage of theoretical content necessary to provide a solid knowledge for a resident doctor?

( ) < 10% ( ) 10-20% ( ) 20-30% ( ) 30-40% ( ) > 40%

7. How many hours a week do you spend reading medical textbooks?

( ) < 1h ( ) 1-3 hs ( ) 3- 4 hs ( ) 4-5 hs ( ) > 5 hs

8. Do you prefer to read printed or digital materials?

( ) printed ( ) digital format

9. Would you rather read classical textbooks or handbooks written for residents?

( ) classical textbooks ( ) handbooks

10. Which digital format books do you own?

11. How many hours a week do you spend reading scientific papers?

( ) < 1h ( ) 1-3 hs ( ) 3- 4 hs ( ) 4-5 hs ( ) > 5 hs

12. Do you have access to the full text of the scientific papers?

( ) yes ( ) no

13. Do you regularly go to the Institution’s library?

( ) yes ( ) no

14. How many hours a week would you have available to participate in preparing cases for clinical forums and discussions?

( ) < 1h ( ) 1-3 hs ( ) 3- 4 hs ( ) 4-5 hs ( ) > 5 hs

3) Strategies for future studies

15. Did you attend a preparatory course to enter the present residency program (basic surgery)?

( ) yes ( ) no

16. Do you intend to attend a preparatory course to enter the next residency program (specialty)?

( ) yes ( ) no

17. What kind of experience have you had with remote learning?

( ) never participated in an e-learning program ( ) participated during high school and undergraduate courses ( ) participated in preparation for admission to the residency program ( ) participated in courses not related to Medicine (music, foreign language etc.)

18. Do you believe that e-learning is an efficient learning strategy?

( ) yes ( ) no

19. Which didactic material you believe is best to be used in an e-learning environment? Please rate from 1 to 10.

( ) classical textbooks excerpts

( ) texts written by the supervisors and directed to the residents

( ) review scientific papers

( ) clinical society guidelines

( ) case reports with comments

( ) classes on video format

( ) operation videos

( ) videos from medical meetings presentations

( ) interactive activities and educational games

( ) clinical case discussion forums

20. Regarding surgical technique, what works best for you?

( ) digital books and atlas ( ) procedural videos

21. Do you need frequent exams to force you to study at a regular interval?

( ) yes ( ) no

22. Which preparatory material are you going to read to prepare for the surgical specialty admission exam?

23. Please, indicate how many regular and mandatory activities are needed regarding an e-learning platform with the goal to organize and stimulate the study process?

4) Interest in having available digital content

24. I believe that my knowledge acquisition is better with on-site classes than reading printed books or e-learning resources.

( ) yes ( ) no

25. Rate how important it is on-site classes available to you.

( ) extremely important ( ) very important ( ) indifferent ( ) irrelevant ( ) extremely irrelevant

26. How many times a month do you believe you would be able to attend such courses during your current residency program.

( ) 1X/month ( ) 2-3X/month ( ) 4-5X/month ( ) more than 6X/month

27. What is the best time for on-site classes in your current residency program?

( ) morning ( ) afternoon ( ) evening

28. Would a video synchronous class be more interesting than an on-site class?

( ) yes ( ) no

29. What would be more interesting for your learning?

( ) on-site classes exclusively ( ) e-learning exclusively ( ) e-learning activities alternated with on-site classes

30. Have you ever used the e-learning Moodle Platform?

( ) yes ( ) no

31. If you did, how would you rate it from 0 to 10?

32. Is the working interface of Moodle interesting?

( ) yes ( ) no

33. Have you ever used other e-learning platforms besides Moodle? If so please name them.

( ) yes ( ) no

## References

[1] Michel JLM, Oliveira RAB, Nunes MPT (2011). Residência Médica no Brasil. Cadernos ABEM.

[2] Santos EG, Ferreira RR, Mannarino VL, Leher EMT, Goldwasser RS, Bravo GP (2012). Cirurgia geral, no centro cirúrgico, comparação entre um hospital universitário e um hospital não universitário. Rev Col Bras Cir.

[3] Botti SHO, Rego S (2010). Processo ensino-aprendizagem na residência médica. Rev Bras Educ Med.

[4] Masseto MT (1985). O processo ensino-aprendizagem no curso de medicina. Rev Fac Educ.

[5] Comissão Nacional de Residência Médica (2006). Resolução n.º 2, de 17 de maio de 2006. Dispõe sobre os requisitos mínimos do Programa de Residência Médica e dá outras providências.

[6] Ruiz JG, Mintzer MJ, Leipzig RM (2006). The impact of e-learning in medical education. Acad Med.

[7] Kim RH, Gilbert T, Ristig K, Chu QD (2013). Surgical resident learning styles faculty and resident accuracy at identification of preferences and impact on ABSITE scores. J Surg Res.

[8] Azer N, Shi X, De Gara C, Karmali S, Birch DW "iBIM" - internet-based interactive modules: an easy and interesting learning tool for general surgery residents (2014). Can. J Surg.

[9] Kotsis SV, Chung KC (2013). Application of the "see one, do one, teach one" concept in surgical training. Plast Reconstr Surg.

[10] Buch SV, Treschow FP, Svendsen JB, Worm BS (2014). Video- or text-based e-learning when teaching clinical procedures A randomized controlled trial. Adv Med Educ Pract.

[11] Ellaway R, Masters K (2008). AMEE Guide 32 e-learning in medical education part 1: learning, teaching and assessment. Med Teach.

[12] Cook DA (2007). Web-based learning pros, cons and controversies. Clin Med (Lond).

[13] McKimm J, Jollie C, Cantillon P (2003). ABC of learning and teaching web based learning. BMJ.

[14] Bamford R, Coulston J (2016). Effective e-learning in surgical education the core values underpinning effective e-learning environments and how these may be enhanced for future surgical education. Ecancermedicalscience.

[15] Yamson GC, Appiah AB, Tsegah M (2018). Electronic vs Print Resources: A survey of perception, usage and preferences among central university undergraduate students. European Scientific Journal.

[16] Leven FJ, Bauch M, Haag M (2006). E-Learning in der Medizinerausbildung in Deutschland Status und Perspektiven. GMS Med Inform Biom Epidemiol.

[17] Oussalah A, Fournier JP, Guéant JL, Braun M (2015). Information-seeking behavior during residency is associated with quality of theoretical learning, academic career achievements, and evidence-based medical practice a strobe-compliant article. Medicine.

[18] Edson RS, Beckman TJ, West CP, Aronowitz PB, Badgett RG, Feldstein DA (2010). A multi-institutional survey of internal medicine residents' learning habits. Medical Teacher.

[19] Larsen DP, Butler AC, Roediger HL (2008). Test-enhanced learning in medical education. Med Educ.

[20] Epstein RM (2011). Assessment in medical education. N Engl J Med.

[21] Silva CS, Souza MB, Silva RS, Medeiros LM, Criado PR (2011). E-learning program for medical students in dermatology. Clinics.

[22] Carmichael M, Reid AK, Karpicke JD (2018). Assessing the impact of educational video on student engagement, critical thinking and learning: the current state of play. Sage Publishing.

[23] Maertens H, Madani A, Landry T, Vermassen F, Van Herzeele I, Aggarwal R (2016). Systematic review of e-learning for surgical training. Br J Surg.

[24] Aryal KR, Pereira J (2014). E Learning in surgery. Indian J Surg.

